# Efficacy of composite versus ceramic inlays and onlays: study protocol for the CECOIA randomized controlled trial

**DOI:** 10.1186/1745-6215-14-278

**Published:** 2013-09-03

**Authors:** Hélène Fron Chabouis, Caroline Prot, Cyrille Fonteneau, Karim Nasr, Olivier Chabreron, Stéphane Cazier, Christian Moussally, Alexandre Gaucher, Inès Khabthani Ben Jaballah, Renaud Boyer, Jean-François Leforestier, Aurore Caumont-Prim, Florence Chemla, Louis Maman, Cathy Nabet, Jean-Pierre Attal

**Affiliations:** 1 Faculté de Chirurgie Dentaire, Université Paris Descartes, Sorbonne Paris Cité, Montrouge 92120, France; 2 Service d’Odontologie, Assistance Publique – Hôpitaux de Paris (AP-HP), Hôpital Charles Foix, Ivry-sur-Seine 94200, France; 3 Ecole doctorale Galillée, Université Paris 13, Sorbonne Paris Cité, Villetaneuse 93430, France; 4 Private Dental Practice, Paris, France; 5 Faculté de Chirurgie Dentaire, Université Paul Sabatier, Toulouse 31062, France; 6 Pôle Odontologie, Hotel-Dieu Saint-Jacques, Toulouse 31059, France; 7 AP-HP, Hôpital Européen Georges-Pompidou (HEGP), Institut National de la Santé et de la Recherche Médicale (INSERM), UMR S872/20, Paris 75015, France

**Keywords:** Dental caries, Inlays, Composite resins, Ceramic, Survival analysis, CAD/CAM, Dental prosthesis, Dental restoration failure, Dental restoration wear

## Abstract

**Background:**

Dental caries is a common disease and affects many adults worldwide. Inlay or onlay restoration is widely used to treat the resulting tooth substance loss. Two esthetic materials can be used to manufacture an inlay/onlay restoration of the tooth: ceramic or composite. Here, we present the protocol of a multicenter randomized controlled trial (RCT) comparing the clinical efficacy of both materials for tooth restoration. Other objectives are analysis of overall quality, wear, restoration survival and prognosis.

**Methods:**

The CEramic and COmposite Inlays Assessment (CECOIA) trial is an open-label, parallel-group, multicenter RCT involving two hospitals and five private practices. In all, 400 patients will be included. Inclusion criteria are adults who need an inlay/onlay restoration for one tooth (that can be isolated with use of a dental dam and has at least one intact cusp), can tolerate restorative procedures and do not have severe bruxism, periodontal or carious disease or poor oral hygiene. The decayed tissue will be evicted, the cavity will be prepared for receiving an inlay/onlay and the patient will be randomized by use of a centralized web-based interface to receive: 1) a ceramic or 2) composite inlay or onlay. Treatment allocation will be balanced (1:1). The inlay/onlay will be adhesively luted. Follow-up will be for 2 years and may be extended; two independent examiners will perform the evaluations. The primary outcome measure will be the score obtained with use of the consensus instrument of the Fédération Dentaire Internationale (FDI) World Dental Federation. Secondary outcomes include this instrument’s items, inlay/onlay wear, overall quality and survival of the inlay/onlay. Data will be analyzed by a statistician blinded to treatments and an adjusted ordinal logistic regression model will be used to compare the efficacy of both materials.

**Discussion:**

For clinicians, the CECOIA trial results may help with evidence-based recommendations concerning the choice of materials for inlay/onlay restoration. For patients, the results may lead to improvement in long-term restoration. For researchers, the results may provide ideas for further research concerning inlay/onlay materials and prognosis.

This trial is funded by a grant from the French Ministry of Health.

**Trial registration:**

ClinicalTrials.gov Identifier: NCT01724827

## Background

The World Health Organization (WHO) estimates the prevalence of dental caries is over 90% among adults worldwide [1,2]. When the loss of tooth substance due to decay is minor, the dentist fills the tooth cavity. With substantial tooth substance loss, the dentist often treats the tooth with a crown, which presents the problem of further destroying the tooth. Large amalgam or build-up amalgam restorations are also used in such cases in many countries; however, amalgam is being abandoned for environmental reasons, especially in Europe [3]. An intermediate technique consists of manufacturing an inlay or onlay for the tooth and this type of restoration has become common because it is a minimally invasive solution (further information on inlays and onlays is available at http://cecoia.fr) [4]. Inlays and onlays can be made of metal alloy, ceramic or composite materials; however, patients tend to refuse metallic restorations for esthetic and financial reasons [5], and thus dentists generally have to choose between composite and ceramic materials.

The chemical composition differs between ceramic and composite inlays and onlays, and explains most of their clinical properties. Ceramic inlays and onlays (ceramics) are mainly composed of glass, with some crystals added to increase strength [6,7]. Composite inlays and onlays (composites) are made of a resinous matrix and fillers of different types [8]. Like glass, ceramics are thus brittle [9] and more prone to fracture than composites [10,11]. However, ceramics are harder than composites: they are thus more wear-resistant but can induce more wear than usual with the opposing tooth’s surface [12]. Furthermore, adhesive cement interfaces are made of composite material, therefore the wear of the interface and restoration material should be closer for composites, with less marginal gaps [13,14]. Another disadvantage of composites is their resinous matrix. An incompletely polymerized matrix can result in monomers than are released into the mouth, which presents some toxicity, whereas ceramics are extremely biocompatible [15-19]. A disadvantage of ceramics is that manufacturing is time-consuming; composites are easier to polish and perhaps less costly.

Some factors may influence the clinical performance of ceramic and composite inlays and onlays differently. Ceramics are resistant to compressive forces but susceptible to shear stresses. Increased compressive forces can be expected with onlays, thus the inlay or onlay factor may influence the performance of the materials differently [10,11]. Bicuspids usually offer more favourable conditions for inlays and onlays than molars: cavities are usually smaller, the effect of masticatory forces and stresses at the adhesive interface are less intense, and access for dental treatment is easier [20]. Tooth type (bicuspid or molar) may thus influence the performance of composite and ceramic inlays and onlays [21]. Tooth vitality may also differently influence the clinical performance of both materials; some *in vitro* studies and simulations have suggested that composites could perform better than ceramics for non-vital teeth [22,23]. Finally, the operator (dentist) who performs the restoration is a key variable [20,24]; practitioners equipped with the computer-assisted design/computer-assisted manufacturing (CAD/CAM) system (CEREC, Sirona Dental Systems, Long Island City, NY, USA) used in this trial manufacture mostly ceramic inlays and onlays, and may require a slight learning curve to manufacture composite inlays or onlays.

A systematic search of the literature conducted for this report identified only two randomized clinical studies that have compared ceramic and composite inlays and onlays (see Research in context section) [25-27]. These studies were small in size (43 and 37 patients) and presented some risk of bias. The results from both trials suggested no clear evidence of a difference between ceramic and composite inlays or onlays. Since then, materials have improved, composites (especially as CAD/CAM blocks) have become much safer and consensus outcomes for evaluating dental restorations have been developed [28].

### Research in context

#### Systematic search of the literature

Following the Cochrane methodology, we searched MED-LINE and Embase for reports of prospective randomized controlled studies comparing at least one composite and one ceramic material for inlay or onlay manufacturing, with a minimum follow-up of 6 months, and without any date or language restriction up to 11 October 2012.

#### Studies identified through the systematic search

*In vitro*: 91 studies

Only one ceramic or one composite (no control or luting agent/base material randomized): 20 studies (27 reports)

Ceramic versus ceramic: three studies

Composite versus composite: two studies

Ceramic versus composite (non-randomized or retrospective study): five studies (eight reports)

Ceramic versus composite (prospective randomized study): two studies (four reports)

#### Interpretation

Only two randomized studies were identified, which compared ceramic and composite materials for inlay or onlay manufacturing. In 2005, a study compared 80 VITA Mark II (ceramic; Vita Zahnfabrik, Bad Säckingen, Germany) and Paradigm (composite; 3M Espe, St Paul, MN, USA) CAD/CAM inlays in 43 adults after 3 years with use of the US Public Health Service (USPHS) modified criteria [29]. The composite inlays performed better for only two items: color match and restoration fracture [25]. In 2006, a study compared 58 CEREC, Vita Dur N (two ceramics; Vita Zahnfabrik), Brilliant DI (Coltene/Whaledent AG, Altstätten, Switzerland) and Estilux (two composites; Heraeus Kulzer GmbH, Hanau, Germany) inlays in 37 patients after 10 years with use of the California Dental Association criteria [30]: survival was similar for all inlays when repairs were not considered failures (75 to 80%) and was better for CEREC ceramic inlays than other inlays when repairs were considered failures (80% versus 51 to 67%) [26]. Data on the material to use for manufacturing inlays or onlays are thus controversial and a RCT is needed.

The main objective of the CEramic and COmposite Inlays Assessment (CECOIA) randomized controlled trial (RCT) is to compare the clinical efficacy of composite and ceramic inlays or onlays for treating moderate substance loss of posterior teeth in adults according to recent consensus outcomes. Secondary objectives include the overall quality, wear and survival of inlays and onlays made of both materials, and prognostic factors of restoration failure, including patient-related items.

## Methods

This trial is a multicenter, randomized, open-label superiority trial with two balanced parallel arms. The trial received approval from the French ethics committee for the protection of persons (Comité de Protection des Personnes (CPP), Ile de France XI, trial number 12029) in May 2012.

### Participants and setting

#### Eligibility criteria for patients

Patients are eligible to participate in the trial if they are adults aged 18 to 70 years, can tolerate restorative procedures and have a posterior moderate-sized dental caries or aged restoration necessitating an inlay or onlay. Exclusion criteria are allergy to one of the materials used, bruxism, severe or acute periodontal or carious disease (greater than or equal to four primary or secondary restorations due to caries in the preceding year) and poor oral hygiene; the tooth to be treated should not need endodontic treatment or retreatment, show mobility >1 mm or a periodontal socket >3 mm or support a removable partial denture.

Patients with a tooth showing a subgingival margin after cavity preparation, that cannot be isolated with use of a rubber dam, or that has cusps that all need to be covered by the restoration are not eligible.

Only one tooth per patient is eligible. If a patient needs more than one inlay/onlay restoration, the tooth with expected cervical limits that are the most coronal, is the eligible tooth. Other required inlays or onlays will be manufactured by the dentist with the usual material (leucite-reinforced glass-ceramic). In case of pulpal exposure, the operator will decide whether a direct pulp capping (with calcium hydroxide) or an endodontic treatment is necessary, randomize the patient after this treatment has been conducted and fill the corresponding fields in the adverse events section of the case report form (CRF).

#### Eligibility criteria for operators (dentists)

Operators will be eligible for inclusion if they have at least 3 years of clinical experience and at least 1 year of experience with chairside CAD/CAM, agree with the intervention protocol, and have no preference for either composite or ceramic to manufacture inlays and onlays.

#### Eligibility criteria for evaluators

Evaluators of restorations during follow-up will be two dentists different from the operators.

#### Setting

Patients will be included and treated in seven centers in France: the dental care departments of two hospitals (Hôpital Charles Foix, Ivry-sur-Seine and Hotel-Dieu Saint-Jacques, Toulouse) and five private practices (four in Paris and one in Lyon). Follow-up data will be collected in these seven centers. Any patient with the eligible criteria visiting one of the included centers will be asked to participate in the study. The consent form can be consulted at http://cecoia.fr: extra section.

### Interventions

Patients will be allocated to receive a leucite-reinforced glass-ceramic or a composite CAD/CAM inlay or onlay.

Among the ceramics currently used, we chose a pressed glass-ceramic because fired feldspathic ceramics have shown higher fracture rates [31], and we chose leucite-reinforced glass-ceramic (IPS Empress CAD, Ivoclar Vivadent, Schaan, Liechtenstein) over lithium disilicate-reinforced glass-ceramic because the latter has been frequently evaluated clinically. Among available composites, we chose a recently developed material (Lava Ultimate, 3 M ESPE, St Paul, MN, USA), which we considered promising after laboratory testing.

Although the purpose was not to study CAD/CAM but to compare composite and ceramic as inlay or onlay materials, we decided to use CAD/CAM for the inlays or onlays in this trial to standardize the manufacturing (as compared with the necessary variability with a dental technician). This technology also simplifies the protocol and conduct of the trial, since some CAD/CAM systems allow for manufacturing inlays or onlays chairside during a single appointment.

For cavity preparation, the operator will choose the color for both evaluated materials (A1/A2/A3). With the patient under local anesthetic, if needed, the cavity will be prepared (for dental caries or former restoration eviction) using a burs sequence (Komet, Rock Hill, SC, USA) specifically designed for the CECOIA trial. Adjacent teeth will be protected (FenderWedge, Directa, Upplands Väsby, Sweden) [32,33]. The following thicknesses will be respected: 2 mm wide and 1.5 mm deep for isthmuses, and 1.2 mm wide for approximal boxes, the approximal overhang not exceeding the box width. Cusps will be covered if the width of the isthmus is greater than half of the intercusp buccolingual distance, the wall is ≤2 mm thick before preparation, the wall is ≤1 mm thick after preparation, the width of the isthmus is close to half the intercusp buccolingual distance and one or more cracks are observed or the preparation is mesio-occlusal-distal or with horizontal forces [34-36]. A base can be applied (dental dam; OptiBond XTR and Premise Flowable, Kerr, Orange, CA, USA).

#### Computer-assisted design/computer-assisted manufacturing (CAD/CAM)

After powder spraying (CEREC Optispray, Sirona Dental Systems), the operator will scan the preparation with use of a digital camera and design the restoration by use of CEREC software (Sirona Dental Systems). If eligibility criteria are still satisfied, the operator will then randomize the tooth to a treatment (randomization procedure described below), insert the corresponding block inside the milling machine and press the button for the restoration to be milled. The operator will then check the approximal contacts of the resulting restoration, correct them if need be, remove the machining lug and weigh the restoration.

#### Surface treatment and polishing of ceramic inlays or onlays

The operator can glaze (IPS Object Fix Putty, glazing paste and stains, Ivoclar Vivadent) or polish the inlay or onlay using the polishers provided in the sequence and diamond paste (OptraFine, Ivoclar Vivadent). The intaglio surface will then be treated with hydrofluoric acid (Porcelain Etchant gel, Bisico, Schaumburg, IL, USA) for 60 seconds, rinsed, dried, silanated (Monobond Plus, Ivoclar Vivadent) and left to dry for at least 3 minutes before sealing.

#### Surface treatment and polishing of composite inlays or onlays

The operator will polish the inlay or onlay using the polishers provided in the sequence, and may modify the color (Kolor Plus, Kerr) of pits and fissures. The intaglio surface will be sandblasted with 50 μm alumina, rinsed, dried, silanated (Monobond Plus) and left to dry for at least 3 minutes before sealing.

#### Inlay or onlay adhesive luting and finishing

A dental dam (DermaDam medium, Bisico) will be used. The tooth surface will be cleaned by air abrasion (RONDOflex, KaVo, Biberach, Germany). Enamel will be etched with orthophosphoric acid (37.5%) for 15 seconds, rinsed thoroughly and dried gently [37]. Adhesive (Optibond XTR) will be applied by gently brushing the tooth surface for 15 seconds, followed by a 3-second air spray and light polymerization of the adhesive for 20 seconds. The inlay will be handled with use of a stick (Stik-N-Place, Directa); adhesive cement (NX3 yellow, Kerr) will be applied generously on the intaglio surface of the restoration. The inlay or onlay will be positioned and maintained. It may be light polymerized for 1 or 2 seconds. Excess cement will be carefully removed by use of dental floss and a curette. Glycerine gel will be applied on the limits of the restoration, followed by light polymerization of the cement for 40 seconds per face. The occlusion will then be adjusted, and the corrected surfaces and cement interface will be polished.

### Outcomes

#### Primary outcome

The primary outcome, clinical efficacy of materials, will be measured by use of the Fédération Dentaire Internationale (FDI) World Dental Federation instrument for assessing dental restorations, described in 2007 [28] and updated in 2010 [38]. This instrument contains three dimensions (18 items): biological (six items), functional (seven items) and esthetic (five items). Each item is assessed by clinical examination on a 5-point Likert scale (1 corresponding to a perfect restoration and 5 corresponding to a restoration that needs to be replaced), and collected in the CRF. All items but one are assessed by the dentist; the remaining item is patient-reported satisfaction. The primary outcome is the worst score for all items (ranging from 1 to 5) at 2-year follow-up (the best material will be the one with the lowest score).

Operators and evaluators, who will assign scores, will be trained in the FDI criteria by means of the e-calib web-based  software (http://zep01793.dent.med.uni-muenchen.de/moodle/) and group training sessions. They will use the evaluation kit specifically designed for evaluating the FDI criteria (EX-KIT 150/250, Deppeler, Rolle, Switzerland).

#### Secondary outcomes

Secondary outcomes will include each item of the FDI instrument, patient-relevant outcomes, quantified wear analysis (through silicone impressions) and overall quality of the restoration (as assessed by dentists). Survival may be evaluated if the follow-up is extended.

#### Follow-up evaluations

The restorations will be evaluated after 1 week by the operator, and after 1 and 2 years by two independent evaluators (Table 1). Follow-up is planned and funded for 2 years; it may be extended to 5 years (as recommended for indirect dental restorations by the FDI) if the grant can be extended.

**Table 1:** Schedule of enrollment, interventions and assessments

	**Study period**				
	**Enrollment**	**Allocation**	**Postallocation**		**Closeout**
Time point	- ≤ 1yr	0	1 wk	1 yr	2 yr
**Enrollment:**					
Eligibility screen	X				
Informed consent	X				
Allocation		X			
**Interventions:**					
(composite or ceramic inlay/onlay)		X			
**Assessments:**					
Baseline variables					
(inlay/onlay, premolar/molar, vital/non vital, operator, sex, date of birth, restoration volume etc.)	X	X			
Outcome variables					
FDI criteria			X	X	X
Radiograph	X		X	X	X
Impression			X	X	X

#### Data collection

Investigators will use a CRF (available at http://cecoia.fr) to record all items required for outcomes analysis. The CRF comprises two adverse events forms (one concerning general health and one concerning inlay/onlay-related events). Patient data will be anonymous because patients will be identified by their inclusion number (the first letter of their first and last name and date of birth only will be registered in the CRF). A clinical research assistant (RB) will visit each center every 20 inclusions to monitor the collection of data (by checking that no CRF field is incomplete) and assess the quality (by comparing the data in the medical record, entered through the online inclusion and randomization software RandoWeb (Assistance Publique – Hôpitaux de Paris (AP-HP), Paris; http://randoweb.aphp.fr), written in the CRF). The data will be entered twice in the database by operators and checked by a data manager (more information about data management procedures is available at http://cecoia.fr: extra and protocole initial sections). Some elements in the CRF allow for checking for operators’ adherence to the protocol.

### Sample size

We estimated the required sample size for the primary outcome (score between 1 and 5, 5 corresponding to the worst score) for the 18 items for each patient. Since the resulting score is an ordinal variable, we used Zhao’s formula, which is based on the expected distribution of responses in each of the five possible ratings [39]. To the best of our knowledge, no data on the FDI score are available. Consequently, we derived assumptions from previous studies [25,26,40-47] that involved the USPHS score [29], with dimensions close to that of the FDI score [28]. Thus, we derived assumptions regarding the expected distribution of ratings for the ceramic and composite groups for each of the three dimensions (biological, functional and esthetic). As a proxy for the FDI score, the worst score across the three dimensions, we estimated the three sample sizes required to guarantee a power of 80%, with a type I error rate of 1.7% (Bonferroni adjustment for three dimensions), to detect expected differences in distribution of ratings between the ceramic and composite groups for each dimension. We considered the largest required sample size, which was found for the biological dimension. Consequently, with an overall type I error risk of 5%, a sample size of 211 patients would guarantee 80% power to detect a difference between an expected 3% for scores 3, 4 or 5 in one group and an expected 7% in the other group. Finally, since several centers and several operators will participate, we expected that outcomes from a same center and a same operator will be more similar than those from different centers or different operators. We took this intracenter/operator correlation of data and applied an inflation factor [48,49], which resulted in an estimated sample size of 358 patients. We will include 400 patients to account for patients lost to follow-up, although we will try to avoid missing data on outcome measures (in particular, by compensating each patient with 100 euros (€100) after 2 years) [50].

The enrolment capacity was estimated to be 75 patients per year for each hospital and 50 patients per year for each private practice. A 1-year period was planned for including these 400 patients.

### Randomization sequence generation

From a literature review, we considered four major factors that could differentially influence the performance of ceramic and composite inlays and onlays (inlay/onlay, premolar/molar, vital/non-vital tooth and operator), and that we should aim for balanced distribution of these factors between the two groups. Consequently, treatment allocation will involve minimization with a 30% random element. Minimization was preferred over stratified randomization from the results of extensive simulations showing minimization with the lowest predictability and imbalance between treatment groups, considering the trial’s sample size and these four factors (details about these simulations and the results are available at http://cecoia.fr) [51,52].

### Allocation concealment

The operator will obtain each randomization allocation through a centralized secured web-based interface that runs the minimization algorithm (RandoWeb). The sequence is thus concealed until the intervention is assigned.

### Implementation

The minimization algorithm was added to the RandoWeb software. It was programmed by an independent statistician. Investigators will enroll participants (inclusion numbers are obtained by use of RandoWeb).

### Blinding/masking

Operators cannot be blinded to the randomization because the intervention differs between both arms (in particular, surface treatments of the upper and intaglio surfaces of the restoration). Moreover, a dentist can easily recognize each material, so neither operators nor evaluators can be blinded. Patients are not blinded, firstly because a few patients had been asked if they would prefer one material to the other and most did not have any preference; secondly because it would complicate the clinical session because the block is inscripted with the name of the material and the intervention differs between both arms; and thirdly because another dentist could tell them if their restoration is made of composite or ceramic.

Therefore, the trial will be open-label. Randomization was thus planned as late as possible to insure that the tooth cavity would be prepared in the same way for both groups and to limit bias due to the absence of blinding. Interventions were standardized as much as possible (in particular, similar adhesive luting procedure) to enhance similarity. The statistician will be blinded to the treatment arms during data analysis.

### Statistical methods

The data will be analyzed by an independent statistician. The unit of analysis will be the patient (only one tooth treated per patient). The demographic and clinical characteristics of patients and treated teeth will be described for both treatment arms with the usual statistics: mean and SD or median and interquartile ranges for quantitative variables, number of subjects, and percentages for qualitative variables [53]. The analyses will be performed according to the intention-to-treat principle [54].

#### Primary outcome analysis

The main analysis will compare the final values of the FDI score (worst score over the three dimensions) between the ceramic and composite groups. The main analysis will be adjusted on the following pre-specified variables: inlay/onlay, premolar/molar, vital/non-vital tooth and operator [53,55]. An ordinal logistic regression model will be used. The operator variable will be modeled as a random effect. The main analysis will take into account missing outcome data by multiple imputation, with the assumption that data are missing at random. We will report the unadjusted analysis as well; that is, the contingency table showing the distribution of FDI scores in the ceramic and composite groups. The distribution of FDI scores will be compared by Fisher’s exact test. All *P* values will be two-tailed, with significance level 0.05.

#### Secondary outcomes analysis

The same analyses will be used to compare both treatments by each of the three dimensions (with an α risk of 1.7% for each dimension). Secondary analyses will also involve FDI items, quantified analysis of wear (by silicone impressions) and analysis of the overall quality of the restoration (assessed by dentists).

#### Subgroup analyses

We will perform subgroup analyses [56] of the following variables: inlay/onlay, premolar/molar, vital/non-vital tooth, inlay/onlay volume, canine or group lateral guidance and occlusal tapping before luting of the inlay/onlay. If interaction tests are performed for six subgroups independent of each other and each at a significance level of 5% (two-sided), the risk of finding at least one false-positive statistically significant interaction (that is, due to sampling fluctuations) is 26% (= 1 − (1 – 0.05)^6^).

## Discussion

For clinicians, the CECOIA trial will help provide evidence-based recommendations concerning the choice of material for inlay/onlay restorations. However, because the manufacturing technique explains part of the inlay/onlay’s properties, the results concerning ceramic and composite inlay/onlay manufacturing will be applicable only for CAD/CAM inlays/onlays and not for traditionally manufactured inlays/onlays. In particular, CAD/CAM composite blocks contain few monomers, which could limit biological failures as compared with traditionally manufactured composites; ceramic blocks present better mechanical properties initially but milling may induce fissures. However, the materials still have a similar composition and this trial may give an idea of their clinical performance.

For patients who receive CAD/CAM inlays/onlays, this trial may lead to an improvement in the longevity of the restorations. For researchers, it may provide ideas for further research concerning the efficacy and prognosis of inlays and onlays.

## Trial status

The trial was submitted for registration at ClinicalTrials.gov on 10 September 2012. Patient recruitment started on 14 September 2012. This protocol was submitted for publication on 20 November 2012. General information about the trial (such as the original protocol submitted to the ethics committee) can be obtained on the trial’s website (http://cecoia.fr). We will share the data obtained.

## Abbreviations

ANSM: Agence Nationale de Sécurité du Médicament et des Produits de Santé; AP-HP: Assistance Publique – Hôpitaux de Paris; CAD/CAM: Computer-assisted design/computer-assisted manufacturing; CECOIA: CEramic and COmposite Inlays Assessment; CONSORT: Consolidated Standards of Reporting Trials; CPP: Comité de Protection des Personnes; CRF: Case report form; DRCD: Département de la Recherche Clinique et du Développement; FDI: Fédération Dentaire Internationale; HEGP: Hôpital Européen Georges-Pompidou; ICH: International Conference on Harmonisation; PHRC: Programme Hospitalier de Recherche Clinique; RCT: Randomized controlled trial; USPHS: US Public Health Service; SPIRIT: Standard Protocol Items: Recommendations for Interventional Trials; WHO: World Health Organization.

## Competing interests

The authors declare that they have no competing interests.

## Authors’ contributions

HFC conceived the study and its design, participated in its coordination, and drafted the protocol in accordance with the International Conference on Harmonisation (ICH) E9 guidelines [57], the Consolidated Standards of Reporting Trials (CONSORT) 2010 statement [58], the CONSORT statement extension for nonpharmacologic treatments [59], the CONSORT statement extension for abstracts [60] and the Standard Protocol Items: Recommendations for Interventional Trials (SPIRIT) 2013 statement [61]. IBJ, RB and ACP participated in the methods development and design of the study. JPA supervised the design and coordination of the study, and the drafting of the protocol. FC, LM and CN provided leadership for the hospitals to participate in the study. SC, CF, AG, CM, CP, KN and OC provided clinical advice. All authors read and approved the final manuscript.

## Authors’ information

HFC teaches dental material courses in Paris and specializes in clinical research; and is the trial’s scientific coordinator. IBJ is the clinical trial coordinator, RB is the clinical research assistant, JFL is the informatics engineer and data manager (head of the Data Monitoring Committee, which is independent from the sponsor and competing interests), and ACP is the statistician. IBJ, RB, JFL and ACP work as methodologists at the clinical research unit, Hôpital Européen Georges-Pompidou (HEGP), Paris. SC, CF, AG, CM, CP, KN and OC are private dental practitioners specializing in direct CAD/CAM. KN and OC work part-time at Hotel-Dieu Saint-Jacques, Toulouse. CN specializes in epidemiology and clinical research; and leads the Toulouse team. LM and FC manage the department of dentistry at the Hôpital Charles Foix, Ivry-sur-Seine; the coordinating center. JPA teaches dental material courses in Paris and works as a private practitioner; and is the trial’s main investigator. HFC, KN, OC, CP, CF, SC, CM and AG are the operators.

## References

[B1] Petersen PE The World Oral Health Report 2003: Continuous Improvement of Oral Health in the 21st century - The Approach of the WHO Global Oral Health Programme 2003 Geneva: WHO 10.1046/j..2003.com122.x 15015736

[B2] Bagramian RA Garcia-Godoy F Volpe AR The global increase in dental caries. A pending public health crisis Am J Dent 2009 22 3 8 19281105

[B3] BIO Intelligence Service Study on the Potential for Reducing Mercury Pollution from Dental Amalgam and Batteries 2012 Paris: BIO Intelligence Service: Final report prepared for the European Commission - DG ENV

[B4] Edelhoff D Sorensen JA Tooth structure removal associated with various preparation designs for posterior teeth Int J Periodontics Restorative Dent 2002 22 241 249 12186346

[B5] McGill S Holmes J The 7/8 crown: a lost art Oper Dent 2012 37 453 457 10.2341/11-402-T 22616922

[B6] Chen X Chadwick TC Wilson RM Hill RG Cattell MJ Crystallization and flexural strength optimization of fine-grained leucite glass-ceramics for dentistry Dent Mater 2011 27 1153 1161 10.1016/j.dental.2011.08.009 21930296

[B7] Lin WS Ercoli C Feng C Morton D The effect of core material, veneering porcelain, and fabrication technique on the biaxial flexural strength and weibull analysis of selected dental ceramics J Prosthodont 2012 21 353 362 10.1111/j.1532-849X.2012.00845.x 22462639

[B8] Drummond JL Degradation, fatigue, and failure of resin dental composite materials J Dent Res 2008 87 710 719 10.1177/154405910808700802 18650540 PMC2561305

[B9] Ansong R Flinn B Chung KH Mancl L Ishibe M Raigrodski AJ Fracture toughness of heat-pressed and layered ceramics J Prosthet Dent 2013 109 234 240 10.1016/S0022-3913(13)60051-7 23566604

[B10] Magne P Belser UC Porcelain versus composite inlays/onlays: effects of mechanical loads on stress distribution, adhesion, and crown flexure Int J Periodontics Restorative Dent 2003 23 543 555 14703758

[B11] Yamanel K Caglar A Gulsahi K Ozden UA Effects of different ceramic and composite materials on stress distribution in inlay and onlay cavities: 3-D finite element analysis Dent Mater J 2009 28 661 670 10.4012/dmj.28.661 20019416

[B12] Mormann WH Stawarczyk B Ender A Sener B Attin T Mehl A Wear characteristics of current aesthetic dental restorative CAD/CAM materials: two-body wear, gloss retention, roughness and Martens hardness J Mech Behav Biomed Mater 2013 20 113 125 23455168 10.1016/j.jmbbm.2013.01.003

[B13] Gladys S Van Meerbeek B Inokoshi S Willems G Braem M Lambrechts P Vanherle G Clinical and semiquantitative marginal analysis of four tooth-coloured inlay systems at 3 years J Dent 1995 23 329 338 10.1016/0300-5712(95)98768-X 8530722

[B14] Krämer N Frankenberger R Leucite-reinforced glass ceramic inlays after six years: wear of luting composites Oper Dent 2000 25 466 472 11203858

[B15] Darmani H Al-Hiyasat AS Milhem MM Cytotoxicity of dental composites and their leached components Quintessence Int 2007 38 789 795 17873986

[B16] Durner J Spahl W Zaspel J Schweikl H Hickel R Reichl FX Eluted substances from unpolymerized and polymerized dental restorative materials and their Nernst partition coefficient Dent Mater 2010 26 91 99 10.1016/j.dental.2009.08.014 19781758

[B17] St John KR Biocompatibility of dental materials Dent Clin North Am 2007 51 747 760 viii 10.1016/j.cden.2007.03.003 17586154

[B18] Wataha JC Rueggeberg FA Lapp CA Lewis JB Lockwood PE Ergle JW Mettenburg DJ In vitro cytotoxicity of resin-containing restorative materials after aging in artificial saliva Clin Oral Investig 1999 3 144 149 10.1007/s007840050093 10803126

[B19] Bakopoulou AA Triviai IN Tsiftsoglou AS Garefis PD In vitro assessment of cytotoxicity of resin-based dental restorative materials on WEHI 13 var fibroblasts Int J Prosthodont 2006 19 13 16 16479751

[B20] Manhart J Scheibenbogen-Fuchsbrunner A Chen HY Hickel R A 2-year clinical study of composite and ceramic inlays Clin Oral Investig 2000 4 192 198 10.1007/s007840000086 11218488

[B21] Manhart J Chen HY Neuerer P Scheibenbogen-Fuchsbrunner A Hickel R Three-year clinical evaluation of composite and ceramic inlays Am J Dent 2001 14 95 99 11507807

[B22] Magne P Knezevic A Simulated fatigue resistance of composite resin versus porcelain CAD/CAM overlay restorations on endodontically treated molars Quintessence Int 2009 40 125 133 19169444

[B23] Magne P Knezevic A Influence of overlay restorative materials and load cusps on the fatigue resistance of endodontically treated molars Quintessence Int 2009 40 729 737 19862399

[B24] Frankenberger R Reinelt C Petschelt A Krämer N Operator vs. material influence on clinical outcome of bonded ceramic inlays Dent Mater 2009 25 960 968 10.1016/j.dental.2009.02.002 19344946

[B25] Fasbinder DJ Dennison JB Heys DR Lampe K The clinical performance of CAD/CAM-generated composite inlays J Am Dent Assoc 2005 136 1714 1723 16383055 10.14219/jada.archive.2005.0116

[B26] Thordrup M Isidor F Hörsted-Bindslev P A prospective clinical study of indirect and direct composite and ceramic inlays: ten-year results Quintessence Int 2006 37 139 144 16475376

[B27] Fron Chabouis H Smail-Faugeron V Attal J Clinical efficacy of composite vs ceramic for inlays and onlays - a systematic review Submitted for publication 10.1016/j.dental.2013.09.009 24119328

[B28] Hickel R Roulet JF Bayne S Heintze SD Mjor IA Peters M Rousson V Randall R Schmalz G Tyas M Vanherle G Recommendations for conducting controlled clinical studies of dental restorative materials. Science Committee Project 2/98–FDI World Dental Federation study design (Part I) and criteria for evaluation (Part II) of direct and indirect restorations including onlays and partial crowns J Adhes Dent 2007 9 1 121 147 18341239

[B29] Bayne SC Schmalz G Reprinting the classic article on USPHS evaluation methods for measuring the clinical research performance of restorative materials Clin Oral Investig 2005 9 209 214 10.1007/s00784-005-0017-0 16421996

[B30] Ryge G Anusavice KJ The California dental association quality evaluation system: a standard for self-assessment Quality Evaluation of Dental Restorations: Criteria for Placement and Replacement 1989 Chicago: Quintessence Publishing 273 290

[B31] Pallesen U van Dijken JW An 8-year evaluation of sintered ceramic and glass ceramic inlays processed by the Cerec CAD/CAM system Eur J Oral Sci 2000 108 239 246 10.1034/j.1600-0722.2000.108003239.x 10872995

[B32] Qvist V Johannessen L Bruun M Progression of approximal caries in relation to iatrogenic preparation damage J Dent Res 1992 71 1370 1373 10.1177/00220345920710070401 1629452

[B33] Lussi A Gygax M Iatrogenic damage to adjacent teeth during classical approximal box preparation J Dent 1998 26 435 441 10.1016/S0300-5712(97)00014-6 9699434

[B34] Krifka S Anthofer T Fritzsch M Hiller KA Schmalz G Federlin M Ceramic inlays and partial ceramic crowns: influence of remaining cusp wall thickness on the marginal integrity and enamel crack formation in vitro Oper Dent 2009 34 32 42 10.2341/08-34 19192835

[B35] Fennis WM Kuijs RH Barink M Kreulen CM Verdonschot N Creugers NH Can internal stresses explain the fracture resistance of cusp-replacing composite restorations?*Eur* J Oral Sci 2005 113 443 448 10.1111/j.1600-0722.2005.00233.x 16202034

[B36] Fennis WM Kuijs RH Kreulen CM Verdonschot N Creugers NH Fatigue resistance of teeth restored with cuspal-coverage composite restorations Int J Prosthodont 2004 17 313 317 15237878

[B37] Fron H Vergnes JN Moussally C Cazier S Simon AL Chieze JB Savard G Tirlet G Attal JP Effectiveness of a new one-step self-etch adhesive in the restoration of non-carious cervical lesions: 2-year results of a randomized controlled practice-based study Dent Mater 2011 27 304 312 10.1016/j.dental.2010.11.006 21122908

[B38] Hickel R Peschke A Tyas M Mjör I Bayne S Peters M Hiller KA Randall R Vanherle G Heintze SD FDI World Dental Federation: clinical criteria for the evaluation of direct and indirect restorations-update and clinical examples Clin Oral Investig 2010 14 349 366 10.1007/s00784-010-0432-8 20628774

[B39] Zhao YD Rahardja D Qu Y Sample size calculation for the Wilcoxon-Mann–Whitney test adjusting for ties Stat Med 2008 27 462 468 10.1002/sim.2912 17487941

[B40] Frankenberger R Taschner M Garcia-Godoy F Petschelt A Krämer N Leucite-reinforced glass ceramic inlays and onlays after 12 years J Adhes Dent 2008 10 393 398 19058686

[B41] Krämer N Taschner M Lohbauer U Petschelt A Frankenberger R Totally bonded ceramic inlays and onlays after eight years J Adhes Dent 2008 10 307 314 18792702

[B42] Guess PC Strub JR Steinhart N Wolkewitz M Stappert CF All-ceramic partial coverage restorations–midterm results of a 5-year prospective clinical splitmouth study J Dent 2009 37 627 637 10.1016/j.jdent.2009.04.006 19477570

[B43] Heintze SD Cavalleri A Forjanic M Zellweger G Rousson V Wear of ceramic and antagonist–a systematic evaluation of influencing factors in vitro Dent Mater 2008 24 433 449 10.1016/j.dental.2007.06.016 17720238

[B44] Manhart J Neuerer P Scheibenbogen-Fuchsbrunner A Hickel R Three-year clinical evaluation of direct and indirect composite restorations in posterior teeth J Prosthet Dent 2000 84 289 296 10.1067/mpr.2000.108774 11005901

[B45] Peumans M De Munck J Van Landuyt K Poitevin A Lambrechts P Van Meerbeek B Two-year clinical evaluation of a self-adhesive luting agent for ceramic inlays J Adhes Dent 2010 12 151 161 20157666 10.3290/j.jad.a17547

[B46] Sjogren G Molin M van Dijken JW A 10-year prospective evaluation of CAD/CAM-manufactured (Cerec) ceramic inlays cemented with a chemically cured or dual-cured resin composite Int J Prosthodont 2004 17 241 246 15119879

[B47] Moncada G Martin J Fernández E Hempel MC Mjör IA Gordan VV Sealing, refurbishment and repair of Class I and Class II defective restorations: a three-year clinical trial J Am Dent Assoc 2009 140 425 432 19339531 10.14219/jada.archive.2009.0191

[B48] Vierron E Giraudeau B Sample size calculation for multicenter randomized trial: taking the center effect into account Contemp Clin Trials 2007 28 451 458 10.1016/j.cct.2006.11.003 17188941

[B49] Haute autorité de Santé Méthodes Quantitatives pour Évaluer les Interventions Visant à Améliorer les Pratiques 2007 Saint-Denis la Plaine: Haute Autorité de Santé

[B50] Fleming TR Addressing missing data in clinical trials Ann Intern Med 2011 154 113 117 10.7326/0003-4819-154-2-201101180-00010 21242367 PMC3319761

[B51] Fron Chabouis H Chabouis F Gillaizeau F Durieux P Chatellier G Ruse ND Attal JP Randomization in clinical trials: stratification or minimization? 2013 Clin Oral Investig: The HERMES free simulation software 10.1007/s00784-013-0949-8 23455573

[B52] Fron H Performances comparées des inlays-onlays composites ou céramiques réalisés par CFAO directe dans le cadre des pertes de substance postérieures moyennes à importantes: essai clinique randomisé multicentrique: élaboration du protocole Master’s thesis 2010: Paris Descartes University

[B53] Austin PC Manca A Zwarenstein M Juurlink DN Stanbrook MB A substantial and confusing variation exists in handling of baseline covariates in randomized controlled trials: a review of trials published in leading medical journals J Clin Epidemiol 2010 63 142 153 10.1016/j.jclinepi.2009.06.002 19716262

[B54] White IR Horton NJ Carpenter J Pocock SJ Strategy for intention to treat analysis in randomised trials with missing outcome data BMJ 2011 342 d40 10.1136/bmj.d40 21300711 PMC3230114

[B55] Yu LM Chan AW Hopewell S Deeks JJ Altman DG Reporting on covariate adjustment in randomised controlled trials before and after revision of the 2001 CONSORT statement: a literature review Trials 2010 11 59 10.1186/1745-6215-11-59 20482769 PMC2886040

[B56] Pocock SJ Assmann SE Enos LE Kasten LE Subgroup analysis, covariate adjustment and baseline comparisons in clinical trial reporting: current practice and problems Stat Med 2002 21 2917 2930 10.1002/sim.1296 12325108

[B57] ICH Steering Committee ICH Harmonised Tripartite Guideline: Statistical principles for clinical trials. International Conference on Harmonisation E9 Expert Working Group Stat Med 1999 18 1905 1942 10532877

[B58] Schulz KF Altman DG Moher D CONSORT Group CONSORT 2010 statement: updated guidelines for reporting parallel group randomized trials Ann Intern Med 2010 152 726 732 10.7326/0003-4819-152-11-201006010-00232 20335313

[B59] Boutron I Moher D Altman DG Schulz KF Ravaud P CONSORT Group Extending the CONSORT statement to randomized trials of nonpharmacologic treatment: explanation and elaboration Ann Intern Med 2008 148 295 309 10.7326/0003-4819-148-4-200802190-00008 18283207

[B60] Hopewell S Clarke M Moher D Wager E Middleton P Altman DG Schulz KF and the CONSORT Group CONSORT for reporting randomised trials in journal and conference abstracts Lancet 2008 371 281 283 10.1016/S0140-6736(07)61835-2 18221781

[B61] Chan AW Tetzlaff JM Altman DG Laupacis A Gøtzsche PC Krleža-Jerić K Hróbjartsson A Mann H Dickersin K Berlin JA Doré CJ Parulekar WR Summerskill WS Groves T Schulz KF Sox HC Rockhold FW Rennie D Moher D SPIRIT 2013 statement: defining standard protocol items for clinical trials Ann Intern Med 2013 158 200 207 10.7326/0003-4819-158-3-201302050-00583 23295957 PMC5114123

